# Sphingolipid-Induced Programmed Cell Death is a Salicylic Acid and EDS1-Dependent Phenotype in Arabidopsis *Fatty Acid Hydroxylase* (*Fah1, Fah2*) and *Ceramide Synthase* (*Loh2*) Triple Mutants

**DOI:** 10.1093/pcp/pcab174

**Published:** 2021-12-15

**Authors:** Stefanie König, Jasmin Gömann, Agnieszka Zienkiewicz, Krzysztof Zienkiewicz, Dorothea Meldau, Cornelia Herrfurth, Ivo Feussner

**Affiliations:** Department for Plant Biochemistry, Albrecht-von-Haller-Institute for Plant Sciences, University of Goettingen, Justus-von-Liebig-Weg 11, Goettingen 37077, Germany; Department for Plant Biochemistry, Albrecht-von-Haller-Institute for Plant Sciences, University of Goettingen, Justus-von-Liebig-Weg 11, Goettingen 37077, Germany; Department for Plant Biochemistry, Albrecht-von-Haller-Institute for Plant Sciences, University of Goettingen, Justus-von-Liebig-Weg 11, Goettingen 37077, Germany; Department for Plant Biochemistry, Albrecht-von-Haller-Institute for Plant Sciences, University of Goettingen, Justus-von-Liebig-Weg 11, Goettingen 37077, Germany; Department for Plant Biochemistry, Albrecht-von-Haller-Institute for Plant Sciences, University of Goettingen, Justus-von-Liebig-Weg 11, Goettingen 37077, Germany; Department for Plant Biochemistry, Albrecht-von-Haller-Institute for Plant Sciences, University of Goettingen, Justus-von-Liebig-Weg 11, Goettingen 37077, Germany; Service Unit for Metabolomics and Lipidomics, Goettingen Center for Molecular Biosciences (GZMB), University of Goettingen, Justus-von-Liebig-Weg 11, Goettingen 37077, Germany; Department for Plant Biochemistry, Albrecht-von-Haller-Institute for Plant Sciences, University of Goettingen, Justus-von-Liebig-Weg 11, Goettingen 37077, Germany; Service Unit for Metabolomics and Lipidomics, Goettingen Center for Molecular Biosciences (GZMB), University of Goettingen, Justus-von-Liebig-Weg 11, Goettingen 37077, Germany; Department of Plant Biochemistry, Goettingen Center for Molecular Biosciences (GZMB), University of Goettingen, Justus-von-Liebig-Weg 11, Goettingen 37077, Germany

**Keywords:** *Arabidopsis thaliana*, Ceramide synthases, Fatty acid hydroxylases, Plant sphingolipids, Programmed cell death, Salicylic acid

## Abstract

Ceramides (Cers) and long-chain bases (LCBs) are plant sphingolipids involved in the induction of plant programmed cell death (PCD). The *fatty acid hydroxylase* mutant *fah1 fah2* exhibits high Cer levels and moderately elevated LCB levels. Salicylic acid glucoside level is increased in this mutant, but no cell death can be detected by trypan blue staining. To determine the effect of Cers with different chain lengths, *fah1 fah2* was crossed with *ceramide synthase* mutants *longevity assurance gene one homologue1-3* (*loh1*, *loh2* and *loh3*). Surprisingly, only triple mutants with *loh2* show cell death detected by trypan blue staining under the selected conditions. Sphingolipid profiling revealed that the greatest differences between the triple mutant plants are in the LCB and LCB-phosphate (LCB-P) fraction. *fah1 fah2 loh2* plants accumulate LCB d18:0, LCB t18:0 and LCB-P d18:0. Crossing *fah1 fah2 loh2* with the salicylic acid (SA) synthesis mutant *sid2-2* and with the SA signaling mutants *enhanced disease susceptibility 1*-*2* (*eds1-2*) and *phytoalexin deficient 4*-*1* (*pad4-1*) revealed that lesions are SA- and EDS1-dependent. These quadruple mutants also confirm that there may be a feedback loop between SA and sphingolipid metabolism as they accumulated less Cers and LCBs. In conclusion, PCD in *fah1 fah2 loh2* is a SA- and EDS1-dependent phenotype, which is likely due to accumulation of LCBs.

## Introduction

Sphingolipids are important molecules with diverse functions in eukaryotic cells. In addition to their structural role as membrane components, in plants they are critical for development and responses to biotic and abiotic stresses (Berkey et al. 2012, Saucedo-Garcia et al. 2015, Ali et al. 2018, Huby et al. 2020). Plant sphingolipids can be divided into four subgroups: free long-chain bases (LCBs), ceramides (Cers), glucosylceramides (GlucCers) and glycosyl inositol phosphoryl ceramides (GIPCs; Pata et al. 2010). Whereas GlucCers and GIPCs are important structural membrane components, Cers and LCBs are mainly described as biosynthetic intermediates and signaling molecules.

LCBs consist of a C18 core with an amino group at C2. Hydroxylation at C1 and C3 generates sphinganine (d18:0), the simplest form of LCB. It can be directly channeled into Cer synthesis or modified by additional hydroxylation at C4 (t18:0) or desaturation (d18:1; Luttgeharm et al. 2016b). Ceramide synthase (CerS) catalyzes the step from LCB to Cer by *N*-acylation of the LCB (Michaelson et al. 2016, Luttgeharm et al. 2016b). The fatty acid moiety can vary in chain length between C16 and C26. In Arabidopsis, there are three different CerS enzymes: Longevity Assurance Gene One (LAG1) Homologue1-3 (LOH1-3; Markham et al. 2011, Ternes et al. 2011). LOH1 and LOH3 preferentially catalyze the reaction with very long-chain fatty acids (VLCFAs) and t18:0 LCBs as substrates (Markham et al. 2011, Ternes et al. 2011, Luttgeharm et al. 2016a). LOH2 catalyzes the reaction preferentially with long-chain fatty acids (LCFAs) of C16 chain length and d18:0 LCB (Markham et al. 2011, Ternes et al. 2011, Luttgeharm et al. 2016a). Consequently, *loh1* mutants accumulate Cers and GlucCers with C16 fatty acid and free trihydroxy sphingoid bases and show lesions late in development, whereas *loh2* mutants show reduced LCFA-containing Cer levels but exhibit no apparent growth phenotype (Markham et al. 2011, Ternes et al. 2011). These data suggest that spontaneous cell death in *loh1* mutants is triggered by the accumulation of either free trihydroxy LCBs or C16 Cer species (Ternes et al. 2011).

The fatty acid moiety of a Cer molecule can be modified by hydroxylation in the alpha position. In Arabidopsis, two fatty acid hydroxylases (FAHs) are described (FAH1 and FAH2; Nagano et al. 2009, König et al. 2012). FAH1 prefers Cers with VLCFAs and FAH2 with C16 fatty acids (Nagano et al. 2012). Hydroxylated Cers (hCers) are found in GlucCers and GIPCs in high amounts (Pata et al. 2010) and might be suppressors of cell death (Townley et al. 2005). In *fah1 fah2* double-mutant plants, which represent a knockdown in the *FAH1* gene and a complete knock out in the *FAH2* gene, however, high amounts of non-hCers in relation to hCers were found but no cell death was observed (König et al. 2012). Additionally, free salicylic acid (SA) and its glucoside salicylic acid glucoside (SAG) accumulate in those plants.

Apart from *loh* and *fah* mutants, deregulation of sphingolipid synthesis in other steps of the pathway often leads to cell death with involvement of SA (Mortimer et al. 2013, Bi et al. 2014, Li et al. 2016, Yanagawa et al. 2017, Zienkiewicz et al. 2020). Different Arabidopsis mutants with defects in sphingolipid metabolic pathways spontaneously accumulate SA and undergo programmed cell death (PCD) (Greenberg et al. 2000, Brodersen et al. 2002, Wang et al. 2008, Mortimer et al. 2013, Bi et al. 2014, Zienkiewicz et al. 2020). So far, the regulation of SA accumulation by sphingolipids on the molecular level is mostly unknown, but there are many hints that Cers and/or LCBs are inducers of PCD signaling and SA induction.

Recently it was shown that ENHANCED DISEASE SUSCEPTIBILITY 1 (EDS1) and PHYTOALEXIN DEFICIENT 4 (PAD4), two key players in regulation of SA synthesis in response to biological and abiotic stresses (Wiermer et al. 2005, Rietz et al. 2011, Cui et al. 2017), are required for Cer-induced cell death in ceramide kinase *accelerated cell death 5* (*acd5*) mutants and LOH2 overexpression lines (Zeng et al. 2021). EDS1 and PAD4 are necessary for promotion of *ISOCHORISMATE SYNTHASE 1 (ICS1)* gene expression and SA induction in Arabidopsis immune responses (Cui et al. 2017). EDS1 is the key signaling protein in the interaction with Toll/interleukin 1 receptor-nucleotide-binding/leucine-rich repeat (TNL) receptors, during effector triggered immunity (ETI) for defense responses and cell death (Wiermer et al. 2005). Together with PAD4 or SENESCENCE-ASSOCIATED GENE101 (SAG101), it acts as a heterodimer to transcriptionally induce defense responses and ICS1-dependent SA synthesis (Rietz et al. 2011, Cui et al. 2017).

In this study, we crossed *fah1 fah2* mutants with *loh* mutants to induce cell death and analyze which components of the sphingolipid pool might be responsible for PCD. We further looked into the involvement of SA in sphingolipid-induced cell death by crossing sphingolipid-deficient mutants with SA biosynthesis or signaling mutants.

## Results

### Crossing *loh2* into *fah1 fah2* mutants leads to lesions in distinct leaf areas

The *fah1 fah2* double mutant has elevated Cer levels and slightly elevated LCB levels, but no severe lesion phenotype (König et al. 2012). To further analyze if Cers with distinct chain lengths may lead to lesions in these mutants, as previous work suggested (Ternes et al. 2011), the double mutant was crossed with the three CerS mutants *loh1, loh2* and *loh3*.

**Fig. 1:**
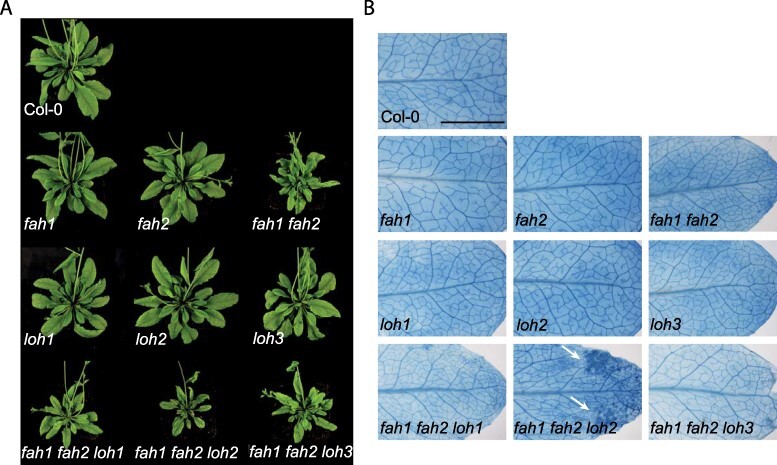
Crosses of *fah1 fah2* double mutants with CerS mutants *loh1–3* lead to a lesion phenotype in *fah1 fah2 loh2* plants. (A) Representative images of single-, double- and triple-mutant plants grown for 35 d under long day conditions. (B) Trypan blue staining of single leaves of 27-day-old plants grown under long day conditions (bar, 5 mm). White arrows show lesions in *fah1 fah2 loh2* plants. Representative leaves out of five replicates are shown.

Crossing *fah1 fah2* with *loh1* and *loh3* produced triple mutants with no additional severe phenotypes. However, crossing *loh2* into *fah1 fah2* produced triple mutants with cell death-derived lesions at distinct areas at the leaves (**Supplementary Fig. S1B**), which was verified by trypan blue staining (**Fig. 1**). Lesion formation in these mutants starts between 14 and 21 d after sowing (**Supplementary Fig. S2**). Additionally, these plants are significantly smaller than the double-mutant plants. Whereas 35-day-old *fah1 fah2* plants are >50% reduced in rosette area compared to Col-0 mutant plants, *fah1 fah2 loh2* plants show <30% of the rosette leaf area of wild-type plants (**Supplementary Fig. S1A**). *fah1 fah2 loh1* and *fah1 fah2 loh3* mutants do not show visible lesions or changes in leaf area compared to *fah1 fah2* grown for up to 35 d under long day conditions.

### *fah1 fah2 loh2* triple mutants accumulate d18:0 and d18:0-P

To reveal the reason for lesion development in *fah1 fah2 loh2* mutant plants, sphingolipid profiles in all mutant lines were compared. Before analyzing large data sets, Cer and SA levels at early (14 days old) and late time points (35 days old) were compared to establish the best plant age for measurements of these metabolites (**Supplementary Fig. S2**). SA levels between Col-0, *fah1 fah2, loh2* and *fah1 fah2 loh2* are similar at both time points, but highest at 35 d. However, changes in Cer and hCer profiles are more distinct in 35-day-old plants. Thus, plants at this age were used for further analysis.

**Fig. 2:**
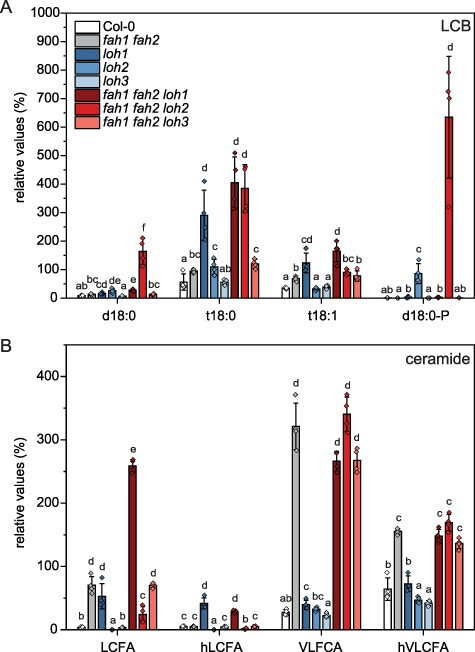
Triple-mutant plants defective in CerS *LOH1* or *LOH2* and both *Fatty Acid Hydroxylases* ( *fah1, fah2*) show altered LCB (A) and Cer (B) levels compared to wild type (Col-0) and to *Fatty Acid Hydroxylase* double mutant ( *fah1 fah2*). Rosette leaves of 35-day-old plants grown under long day conditions were extracted and analyzed. LCFA, Cers with C16–C18 FA moiety; hLCFA, Cers with C16–C18 α-hydroxylated FA moiety; VLCFA, Cers with C20–C28 FA moiety; hVLCFA, Cers with C20–C28 α-hydroxylated FA moiety. Relative values were calculated according to 100% of wild-type peak area of the total LCB or Cer signals. Values represent the mean ± SD of four biological replicates (*n* = 4). The experiment was repeated once with similar tendencies. Statistical analysis was performed with log-transformed data by one-way analysis of variance (ANOVA) with Tukey’s post hoc test (*P *< 0.05). Different letters indicate significant differences with *P *< 0.05.

Analysis of all mutant lines revealed that the most obvious changes between the triple mutants are in their LCB profiles. As shown in earlier studies, changes in LCB levels in the *fah1 fah2* double mutant are only minor (1.5–2-fold increase) compared to Col-0. However, *fah1 fah2 loh2* triple mutants accumulate d18:0 (20-fold compared to Col-0) and d18:0-P (not detectable in Col-0) as well as t18:0 (7-fold compared to Col-0; **Fig. 2A**). In contrast, in *fah1 fah2 loh1* triple mutants, t18:0 (7-fold compared to Col-0) and t18:1 (4.7-fold) are the dominant enriched species, but the amount is comparable to the *loh1* single mutant. *fah1 fah2 loh3* triple mutants do not show significant changes compared to *fah1 fah2* mutant LCB levels. Although elevated levels of t18:0 and t18:1 LCBs could be detected in the different mutants, no phosphorylated versions of these species could be detected. In addition, d18:1 levels were not detected as well.

In addition to LCBs, Cers are suspected to be involved in cell death induction in leaves. Changes in Cer profiles are especially visible in the *fah1 fah2 loh1* triple mutant (**Fig. 2B**). Compared to the *fah1 fah2* mutant, LCFA (C16-C18)-containing Cers increased up to three times, whereas in the *fah1 fah2 loh2* triple mutant, these species are strongly reduced. VLCFA-containing Cers are comparable in the double and triple mutants.

### SAG levels are highly elevated in mutants with *fah1 fah2* background

**Fig. 3:**
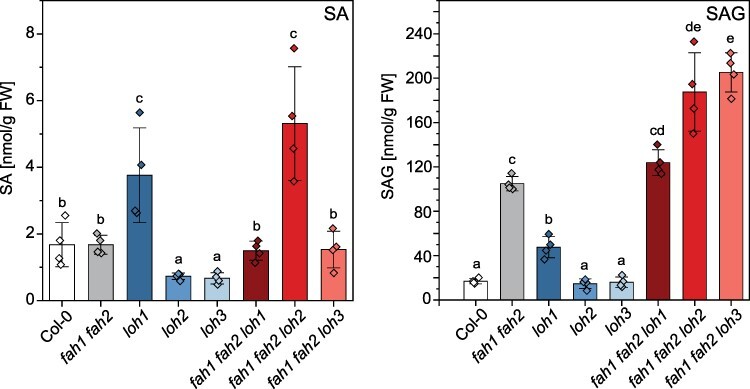
SA (A) and SAG (B) levels are altered in CerS (*loh1, loh2, loh3*) and *Fatty Acid Hydroxylase* ( *fah1, fah2*) mutant plants and crosses. Rosette leaves of 35-day-old plants grown under long day conditions were extracted and analyzed. Values represent the mean ± SD of four biological replicates (*n* = 4). The experiment was repeated once with similar tendencies. Statistical analysis was performed with log-transformed data by one-way analysis of variance with Tukey’s post hoc test (*P *< 0.05). Different letters indicate significant differences with *P *< 0.05.

As SA is described to be involved in induction of sphingolipid-derived cell death, SA and SAG were measured in rosette leaves. Among all mutant lines tested, the highest SA levels are detectable in the *fah1 fah2 loh2* mutant (5.3 nmol/g fresh weight [FW]) followed by the *loh1* single mutant with 3.8 nmol/g FW (**Fig. 3A**). Compared to 1.7 nmol/g FW in Col-0, the induction is minor. However, SAG strongly accumulates in the *fah1 fah2* double mutant (105 nmol/g FW) and in all three triple mutant lines (*fah1 fah2 loh1*: 124 nmol/g FW, *fah1 fah2 loh2*: 188 nmol/g FW, *fah1 fah2 loh3*: 205 nmol/g FW) compared to Col-0 (17 nmol/g FW; **Fig. 3B**).

### Reduction of SA levels by disruptions in SA synthesis or signaling represses the lesion phenotype of the *fah1 fah2 loh2* mutant

To connect SA accumulation with the lesion phenotypes, mutants were crossed with the SA biosynthesis mutant *sid2-2* (defective in *ISOCHORISMATE SYNTHASE 1*) and additionally with *ENHANCED DISEASE SUSCEPTIBILITY1-2* (*eds1-2*) and *PHYTOALEXIN DEFICIENT4* (*pad4-1*). *eds1-2* and *pad4-1* are signaling mutants defective in the induction of SA synthesis by ETI (Cui et al. 2017).

**Fig. 4:**
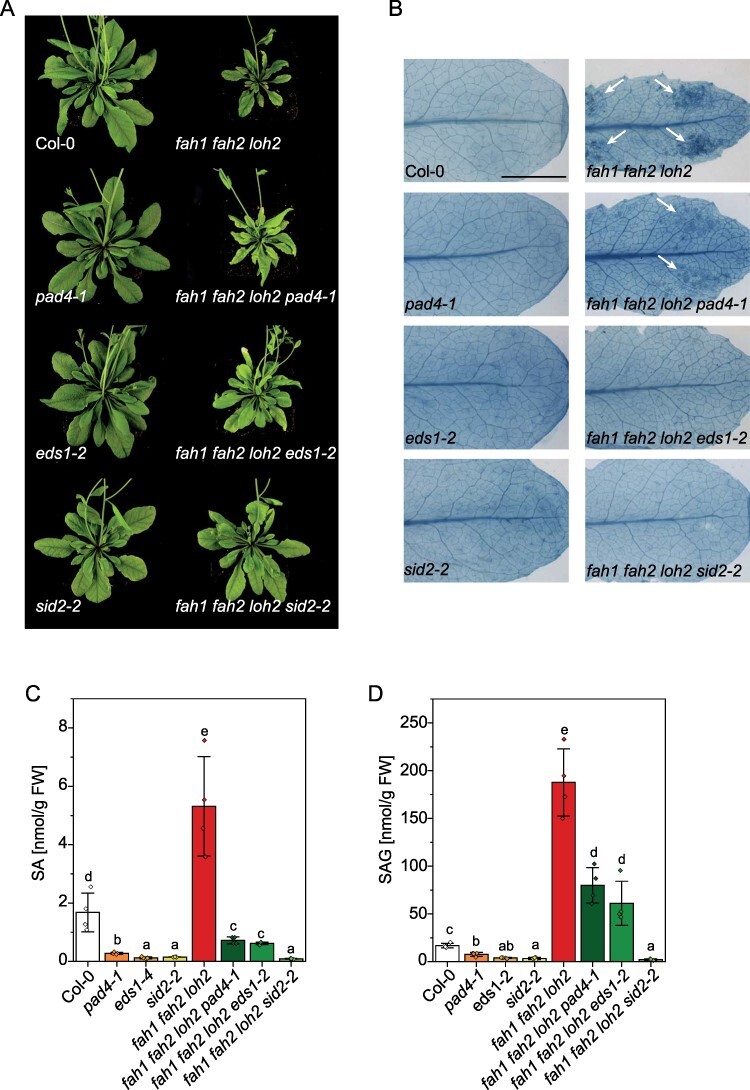
Crosses of *fah1 fah2 loh2* triple mutants with SA synthesis (*sid2-2*) and signaling mutants (*eds1-2, pad4-1*) show changes in lesion phenotype. (A) Representative images of single-, triple- and quadruple-mutant plants grown for 35 d under long day conditions. (B) Trypan blue staining of single leaves of 25-day-old plants grown under long day conditions (bar, 5 mm). White arrows show lesions in *fah1 fah2 loh2* and *fah1 fah2 loh2 pad4-1* plants. Representative leaves out of four replicates are shown. The experiment was repeated twice with similar results. (C, D) SA and SAG levels of rosette leaves of 35-day-old plants. Mean values ± SD of four biological replicates (*n* = 4) are shown. The experiment was repeated once with similar tendencies. Statistical analysis was performed with log-transformed data by one-way analysis of variance with Tukey’s post hoc test (*P *< 0.05). Different letters indicate significant differences with *P *< 0.05.

After crossing the *fah1 fah2 loh2* triple mutant with *pad4-1*, the lesion phenotype detected by trypan blue staining is still visible but less prominent than in *fah1 fah2 loh2* mutant plants (**Fig. 4B**). In contrast to this, in crossings of *fah1 fah2 loh2* triple mutant with *eds1-2* or *sid2-2*, no lesions can be detected. Rosette leaf areas of all quadruple mutants are smaller than the respective single mutant ( *fah1 fah2 loh2 pad4-1*: 58%, *fah1 fah2 loh2 eds1-2*: 46%, *fah1 fah2 loh2 sid2-2*: 34%), but size reduction is not as strong as for the *fah1 fah2 loh2* mutant compared to Col-0 (73%; **Fig. 4A**, **Supplementary Fig. S1**). Especially *fah1 fah2 loh2 sid2-2* plants show only a small reduction in size and resemble the phenotype of the *sid2-2* single mutant (**Fig. 4A**, **Supplementary Fig. S1**).

To confirm the reduction of SA and SAG in the mutants, these phytohormones were measured in all mutant lines. SA levels in the *fah1 fah2 loh2* plants crossed with *eds1-2* and *pad4-1* are lower than wild-type levels. SAG is still induced in those plants, but at a lower level than in *fah1 fah2 loh2* plants ( *fah1 fah2 loh2 pad4-1*: 43% of *fah1 fah2 loh2* levels, *fah1 fah2 loh2 eds1-2:* 33% of *fah1 fah2 loh2* levels; **Fig. 4C**). *fah1 fah2 loh2* plants crossed with *sid2-2* plants show no SA or SAG accumulation. In addition to the *fah1 fah2 loh2* triple mutant, the *fah1 fah2* double mutant was also crossed with *sid2-2* to analyze which phenotypic changes are due to SA accumulation in those plants. Also in this mutant line, no SA or SAG is detectable (**Supplementary Fig. S4**).

**Fig. 5:**
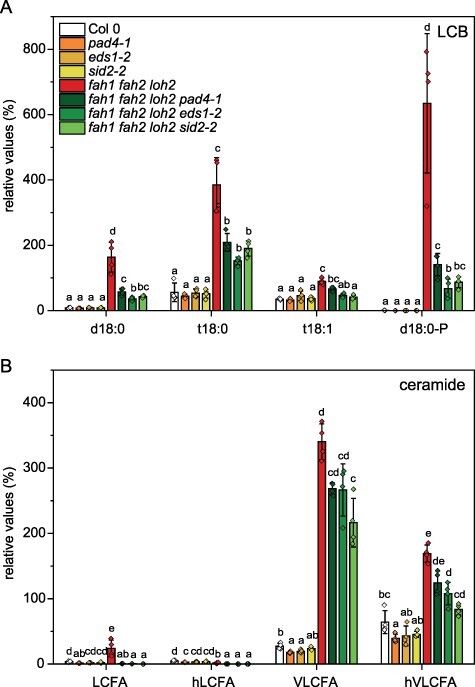
LCB (A) and Cer (B) profiles are altered in crosses of *fah1 fah2* double and *fah1 fah2 loh2* triple mutants with SA synthesis (*sid2-2*) and signaling mutants (*eds1-2, pad4-1*). Rosette leaves of 35-day-old plants grown under long day conditions were extracted and analyzed. LCFA, Cers with C16–C18 FA moiety; hLCFA, Cers with C16–C18 α-hydroxylated FA moiety; VLCFA, Cers with C20–C28 FA moiety; hVLCFA, Cers with C20–C28 α hydroxylated FA moiety. Relative values were calculated according to 100% of wild-type peak area of the total LCB or Cer signal. Values represent the mean ± SD of four biological replicates (*n* = 4). The experiment was repeated once with similar tendencies. Statistical analysis was performed with log-transformed data by one-way analysis of variance with Tukey’s post hoc test (*P *< 0.05). Different letters indicate significant differences with *P *< 0.05.

Sphingolipid analyses of the mutants show that defects in SA synthesis and/or signaling also have an influence on the sphingolipidome of the plants. All SA mutant crosses showed a reduction of LCB accumulation relative to the *fah1 fah2 loh2* triple mutant. d18:0 and d18:0-P are especially strongly reduced in the quadruple mutants (**Fig. 5A**). Additionally, LCFA-containing Cers are depleted in the quadruple mutants, and reductions of VLCFA-containing Cer are only minor (**Fig. 5B**). In contrast to this, *fah1 fah2* mutants crossed with *sid2-2* show a strong reduction in VLCFA-containing Cers and only a slight reduction in LCB levels compared to *fah1 fah2* (**Supplementary Fig. S4**). In contrast to LCBs and Cers, levels of GlucCer and GIPC are not changed due to disruption of SA synthesis (**Supplementary Fig. S3**).

## Discussion

Accumulation of LCBs and/or Cers is suspected to be responsible for sphingolipid-induced cell death in several mutants with disruptions in the sphingolipid pathway (Berkey et al. 2012, Bi et al. 2014, Yanagawa et al. 2017, Zienkiewicz et al. 2020). However, *fah1 fah2* mutant plants exhibit strong accumulations of non-hCers and hydroxylated very long-chain fatty acid (hVLCFA)-containing Cers, moderate increases in LCB levels and enhanced SA levels, but no lesion phenotype (König et al. 2012). Therefore, the intention behind crossing *fah1 fah2* plants with *loh* plants was to determine if specific changes in the sphingolipidome of those mutants could be correlated with the induction of PCD. *fah1 fah2* mutants were crossed with *loh1, loh2* and *loh3* mutants to shift the Cer accumulation to VLCFA Cer or C16 Cer. C16 Cers are discussed as PCD-inducing sphingolipids in *loh1* plants under certain conditions (Ternes et al. 2011) and in *LOH2*-overexpressing plants (Luttgeharm et al. 2015). Unexpectedly, *fah1 fah2* crosses with *loh2*, but not those with *loh1*, produced triple mutants with PCD (**Figs. 1** and **2**). In *fah1 fah2 loh2* plants, LCFA-containing Cers are strongly reduced compared to *fah1 fah2*, but this is also more pronounced in the *loh2* single mutant. Therefore, it seems that the other main difference in the sphingolipid profile of these plants, the elevated levels of d18:0 LCB and d18:0-P LCB, is most likely the underlying reason for the lesion phenotype. LCBs are also suspected to be involved in PCD induction upon Fumonisin B_1_ treatment, a fungal toxin inhibiting LOH1 (Luttgeharm et al. 2016a). LOH1 inhibition induces a strong accumulation of d18:0 and t18:0 as well as LCFA-containing Cer species. This is supported by the analysis of mutants defective in different steps of the sphingolipid pathway or pathogen-infected plants, which collectively suggest that accumulation of LCBs induces cell death in plants (Shi et al. 2007, Peer et al. 2010, Saucedo-Garcia et al. 2015, Luttgeharm et al. 2016a, Yanagawa et al. 2017). The different roles of d18:0 and t18:0 LCBs are still not clear. In case of *fah1 fah2 loh2*, both species are induced, but compared with *fah1 fah2 loh1* accumulation of d18:0 LCB is specific to the former. d18:0 LCB may therefore be responsible for the differences in lesion development between the mutants. This scenario is supported by reports from other sphingolipid mutants like the *serine palmitoyltransferase* mutant (*lcb2a*; Saucedo-García et al. 2011) and the *sphingoid base hydroxylase* double mutant (*sbh1-1 sbh2-1*; Chen et al. 2008), which show increased d18:0 contents in combination with PCD. Nevertheless, it could also be that d18:0 LCB is acting together with t18:0 LCB and that the combined accumulation of both LCBs passes a critical level that leads to induction of PCD. LCB-phosphates (LCB-Ps) are thought to be the inactivated form of LCB that do not induce PCD and do not influence LCB-induced cell death (Yanagawa et al. 2017, Glenz et al. 2019). The high accumulation of d18:0-P in *fah1 fah2 loh2* plants might be due to the partial removal of d18:0 by phosphorylation by LCB kinase to avoid the harmful effect of the molecule (Imai and Nishiura 2005).

LOH2 seems not to be essential under normal growth conditions, as *loh2* mutants do not show a phenotype compared to wild type (Markham et al. 2011, Ternes et al. 2011, Luttgeharm et al. 2015). However, overexpression of *LOH2* leads to dwarfed plants with high amounts of LCFA-containing Cers and to higher resistance to FB_1_ treatment (Luttgeharm et al. 2015). Therefore, the authors suspected that LOH2 might be a safety valve for plants to sequester excess LCBs. In the case of the *fah1 fah2* double mutant, LOH2 might be essential to channel excess LCBs into the Cer pool to avoid toxic LCB levels in the plant. In triple mutants with *loh1* or *loh3*, activity of LOH2 and the other remaining LOH are high enough not to exceed the critical level of LCBs. In the *fah1 fah2 loh2* mutant, this safety valve is not functional anymore as Cer synthesis of C16 fatty acids with d18:0 LCB is blocked and d18:0 LCB accumulate. Excess d18:0 can then be partially removed by phosphorylation resulting in accumulation of d18:0-P. It might be interesting to determine if blocking phosphorylation, e.g. in LCB kinase mutants, would lead to a more severe phenotype.

All mutants with *fah1 fah2* background showed high levels of total SA. To test the role of SA in the development of lesions and reduction in rosette size, mutants were crossed with the SA synthesis mutant *sid2-2* (isochorismate synthase 1 deficient). Additionally, as it is known that sphingolipid metabolism in plants is connected to plant defense (Huby et al. 2020), plants were crossed with *eds1-2* and *pad4-1.* The same SA-mutant set was recently used by Zeng and colleagues to analyze the effect of SA and SA signaling on cell death in *acd5* mutants and LOH2 overexpression mutants (Zeng et al. 2021). They could show that EDS1 and PAD4 contribute to Cer-mediated accumulation of SA and cell death in both transgenic lines. In our study, the lesion phenotype of *fah1 fah2 loh2* was eliminated in *fah1 fah2 loh2 sid2-2* and *fah1 fah2 loh2 eds1-2* mutants but still present in *fah1 fah2 loh2 pad4-1* although at a less severe level (**Fig. 3**), showing that the phenotype is dependent on SA and EDS1 but less on PAD4. Lapin and colleagues showed that EDS1 heterodimerizes with SAG101 and N REQUIRED GENE1 (NRG1) to promote TNL-dependent cell death in Arabidopsis, and that this strong cell death activity does not occur when PAD4 substitutes for SAG101 (Lapin et al. 2019). EDS1 and PAD4 instead mediate transcriptional promotion of defense reactions important for limiting bacterial growth. The distinction between both pathways seems not to be very strict and a small proportion of cell death can account from EDS1–PAD4 signaling. This could explain why no cell death is visible in *fah1 fah2 loh2 eds1-2* mutants but is still found in *fah1 fah2 loh2 pad4-1* mutants.

Interestingly, not only the phenotype of the plants changed due to SA mutations but also the sphingolipid profile (**Fig. 5**). In *fah1 fah2 loh2 eds1-2, fah1 fah2 loh2 pad4-1* and especially in *fah1 fah2 loh2 sid2-2* plants, the accumulation of Cers and LCBs is significantly reduced, showing that SA level has an influence on the sphingolipidome of Arabidopsis. Indeed, Shi et al. (2015) showed that SA influences sphingolipid fluxes in Arabidopsis seedlings by in silico flux balance analysis and suggested SA could also act upstream of LCB or Cer signals (Shi et al. 2015). Interestingly, Zeng and colleagues could show that primarily EDS1 and only to a much lesser extent PAD4 are involved in regulation of Cer and LCB metabolism (Zeng et al. 2021). Cer and LCB accumulation in *acd5* mutants and in LOH2 overexpression lines was strongly reduced only in crossings with *eds1-2*. Our data therefore only partly confirm these results that SA levels in the plant influence LCB and Cer levels and that there might be a feedback loop between SA signaling and sphingolipid biosynthetic pathways.

Together, our data strongly suggest that lesion formation as a result of changes in sphingolipid metabolism in *fah1 fah2 loh2* plants is SA-dependent and transduced via EDS1 signaling. SA signaling itself influences the accumulation of Cers and LCBs in Arabidopsis, suggesting a positive feedback loop between both pathways.

## Material and Methods

### Plant material

The *Arabidopsis thaliana* plants used in this study were of the Columbia (Col-0) ecotype background. The seeds of the T-DNA insertion mutants—*fah1* (SALK_140660), *fah2* (SAIL_862_H01), *loh1* (SALK_069253), *loh2* (SALK_018608) and *loh3* (SALK_150849)—were obtained from the Nottingham Arabidopsis Stock Centre. All mutants are knockout mutants except *fah1* that is a knock-down mutant (Ternes et al. 2011, König et al. 2012). The *sid2-2*, *eds1-2* and *pad4-1* mutants were kindly provided by Prof. Christiane Gatz (University of Goettingen, Germany). Double, triple and quadruple mutants were generated by crossing the above mutants. The homozygous insertion of T-DNA was verified by polymerase chain reaction analysis performed on genomic DNA. Primer sequences used for genotyping are provided in **Supplementary Table S1**. For soil-grown plants, Arabidopsis seeds were sown on soil and then stratified at 4°C for 2 d. Plants were grown at 22°C under long day conditions (16 h light : 8 h dark), with a light intensity of 120–150 μmol m^−2^ s^−1^.

### Trypan blue staining

To visualize the cell death phenotype, trypan blue staining was performed as described previously (Fernández-Bautista et al. 2016) and analyzed using a stereomicroscope Olympus SZX12 (Olympus, Hamburg, Germany).

### Quantification of SA and SAG

For absolute quantification of SA and SAG, 100 mg of frozen homogenized rosette leaf tissue was extracted with methyl-*tert*-butyl ether together with 10 ng D4-SA (C/D/N Isotopes Inc., Pointe-Claire, Canada), reversed-phase-separated using an ACQUITY UPLC^®^ system (Waters Corp., Milford, MA, USA) and analyzed by nanoelectrospray ionization (nanoESI) (TriVersa Nanomate^®^; Advion BioSciences, Ithaca, NY, USA) coupled with an AB Sciex 4000 QTRAP^®^ tandem mass spectrometer (AB Sciex, Framingham, MA, USA) employed in scheduled multiple reaction monitoring mode (Herrfurth and Feussner 2020). The reversed-phase separation of SA and SAG was achieved by UPLC using an ACQUITY UPLC^®^ HSS T3 column (100 mm × 1 mm, 1.8 μm; Waters Corp., Milford, MA, USA). Solvents A and B were water and acetonitrile/water (90 : 10, *v/v*), respectively, both containing 0.3 mmol/l NH_4_HCOO (adjusted to pH 3.5 with formic acid). Mass transitions and optimized parameters for the detection of SA and SAG were as follows: 137/93 [declustering potential (DP) 25 V, entrance potential (EP) 6 V, collision energy (CE) 20 V] for SA, 141/97 (DP 25 V, EP 6 V, CE 22 V) for D_4_-SA and 299/137 (DP 30 V, EP 4 V, CE 18 V) for SAG. Quantification was carried out using a calibration curve of intensity (*m/z*) ratios of [unlabeled]/[deuterium-labeled] *vs*. molar amounts of unlabeled (0.3–1,000 pmol). Data can be found in Supplemental Dataset II.

### Sphingolipid analysis

The analysis of LCBs, LCB-Ps, Cers, GlucCers and GIPCs was performed according to a method previously described (Zienkiewicz et al. 2020). For extraction of sphingolipids, 100 mg of frozen homogenized rosette leaf tissue was resuspended in propan-2-ol/hexane/water (60 : 26 : 16, *v*/*v*/*v*) and incubated at 60°C for 30 min (Grillitsch et al. 2014). After centrifugation at 635×*g* for 20 min, the supernatant was dried under a nitrogen stream and dissolved in tetrahydrofuran/methanol/water (4 : 4 : 1, *v*/*v*/*v*). For analysis of LCB-Ps, the resulting extract was measured after acetyl derivatization (Yanagawa et al. 2017). Sphingolipids were analyzed, and resulting analytical data were processed as previously described with some modifications (Tarazona et al. 2015). The lipid extracts were reversed-phase-separated using an ACQUITY UPLC^®^ system (Waters Corp., Milford, MA, USA) and analyzed by nanoESI (TriVersa Nanomate^®^; Advion BioSciences, Ithaca, NY, USA) coupled with an AB Sciex 6500 QTRAP^®^ tandem mass spectrometer (AB Sciex, Framingham, MA, USA) employed in scheduled multiple reaction monitoring mode. The reversed-phase separation was achieved by UPLC using an ACQUITY UPLC^®^ HSS T3 column (100 mm × 1 mm, 1.8 μm; Waters Corp., Milford, MA, USA). Solvents A and B were methanol/20 mM ammonium acetate (3 : 7, *v/v*) and tetrahydrofuran/methanol/20 mM ammonium acetate (6 : 3 : 1, *v/v/v*), respectively, both containing 0.1%, *v/v* acetic acid. Data can be found in Supplemental Dataset I.

## Supplementary Material

pcab174_Supp

## Data Availability

All source data of this paper are provided as supplemental data sets in the paper.
