# Supplementary material for: Sphingolipid-Induced Programmed Cell Death is a Salicylic Acid and EDS1-Dependent Phenotype in Arabidopsis Fatty Acid Hydroxylase (Fah1, Fah2) and Ceramide Synthase (Loh2) Triple Mutants
Source: Plant Cell Physiol. 2021 Dec 15;63(3):317–25. doi: 10.1093/pcp/pcab174 (PMC8917834; doi:10.1093/pcp/pcab174)
Supplement: pcab174_Supp [file pcab174_supp.zip › pcp-2021-e-00389-File011.pdf]

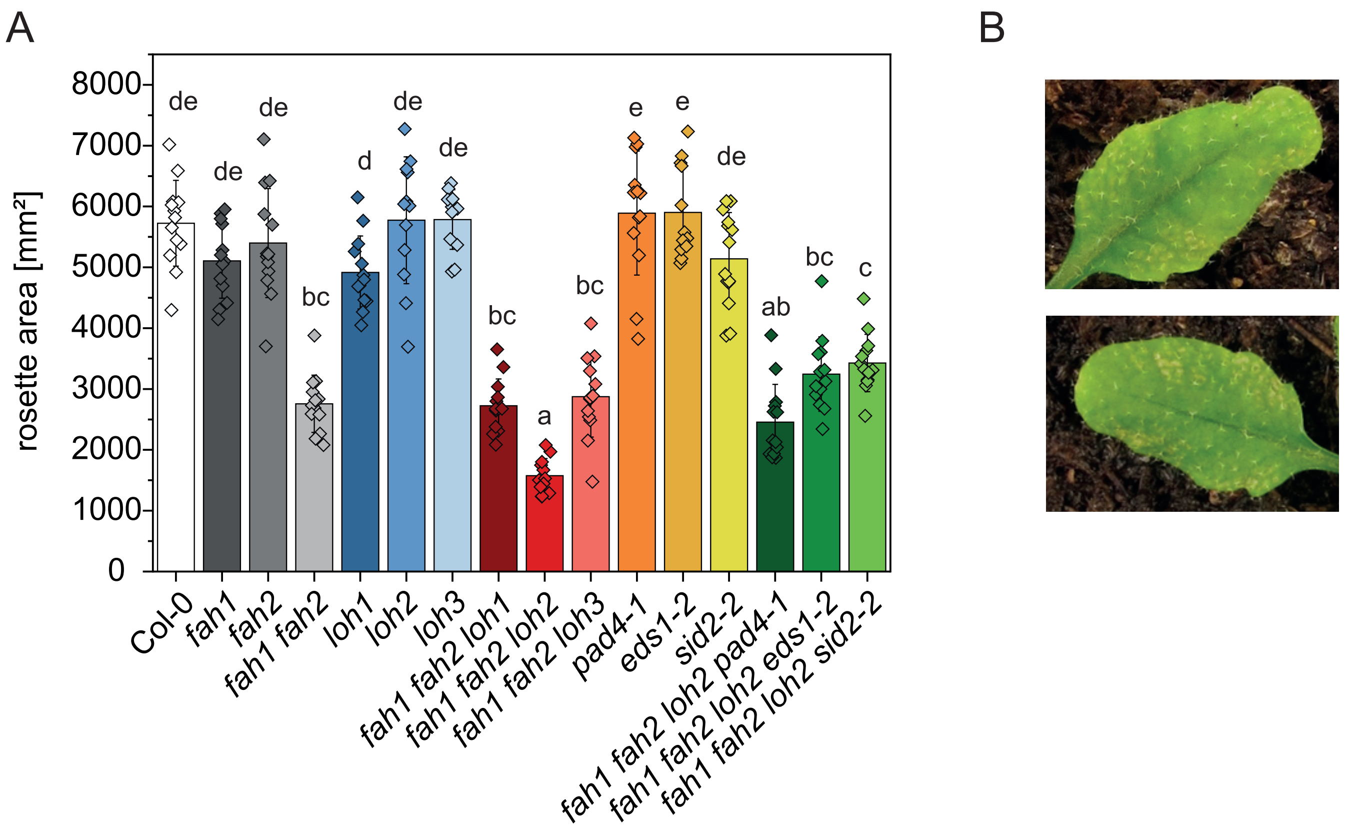

Fig. S1 (A) Crossing SA synthesis and signaling mutants with plants defective in Fatty Acid Hydroxylase1 and 2 and CerS LOH2 partially complements rosette area reduction. Rosette area was quantified from 35-day-old plants grown under long day conditions. Pictures of each plant were taken from above and the projected rosette area was determined by a software program provided by datInf (Tübingen). Values represent the mean ±SD of 13 biological replicates out of two independent experiments. Statistical analysis was performed by one-way analysis of variance (ANOVA) with Tukey's post hoc test ($P<0.05$). Different letters indicate significant differences with $P<0.05$. (B) Visible lesions in *fah1 fah2 loh2* mutant plants.
