# Supplementary material for: Sphingolipid-Induced Programmed Cell Death is a Salicylic Acid and EDS1-Dependent Phenotype in Arabidopsis Fatty Acid Hydroxylase (Fah1, Fah2) and Ceramide Synthase (Loh2) Triple Mutants
Source: Plant Cell Physiol. 2021 Dec 15;63(3):317–25. doi: 10.1093/pcp/pcab174 (PMC8917834; doi:10.1093/pcp/pcab174)
Supplement: pcab174_Supp [file pcab174_supp.zip › pcp-2021-e-00389-File012.pdf]

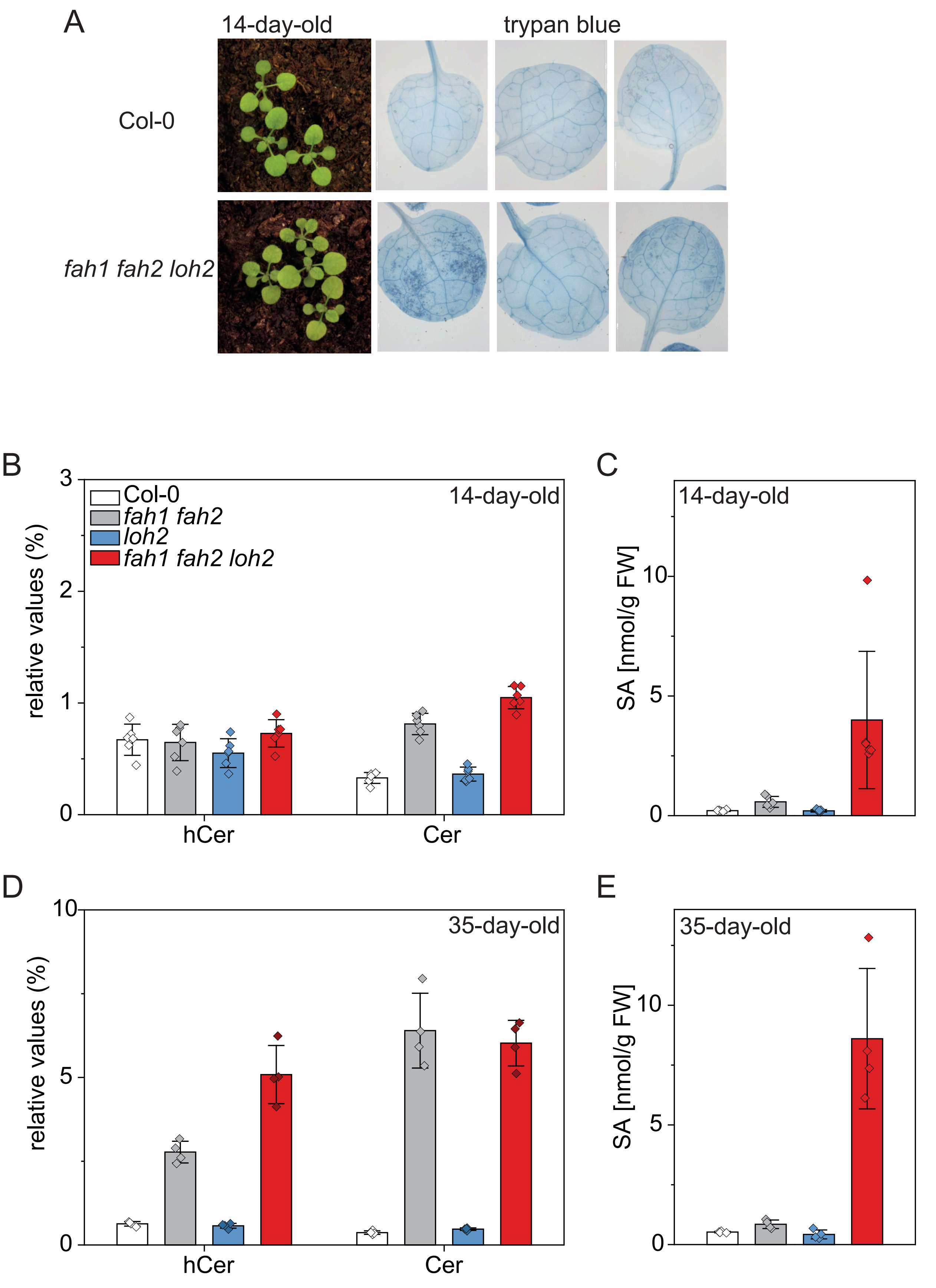

Fig. S2 (A) Col-0 and *fah1 fah2 loh2* 14-day-old plants grown under long day conditios. Trypan blue staining was used for detection of cell death. (B-E) SA , Cer and hCer content in 14- and 35-day-old plants. Rosette leaves of plants grown under long day conditions were extracted and analyzed. Values represent the mean ±SD of six (14-day-old) or four (35-day-old) biological replicates (n=6 and n=4). The experiment was repeated once with similar tendencies.
