# Supplementary material for: Sphingolipid-Induced Programmed Cell Death is a Salicylic Acid and EDS1-Dependent Phenotype in Arabidopsis Fatty Acid Hydroxylase (Fah1, Fah2) and Ceramide Synthase (Loh2) Triple Mutants
Source: Plant Cell Physiol. 2021 Dec 15;63(3):317–25. doi: 10.1093/pcp/pcab174 (PMC8917834; doi:10.1093/pcp/pcab174)
Supplement: pcab174_Supp [file pcab174_supp.zip › pcp-2021-e-00389-File013.pdf]

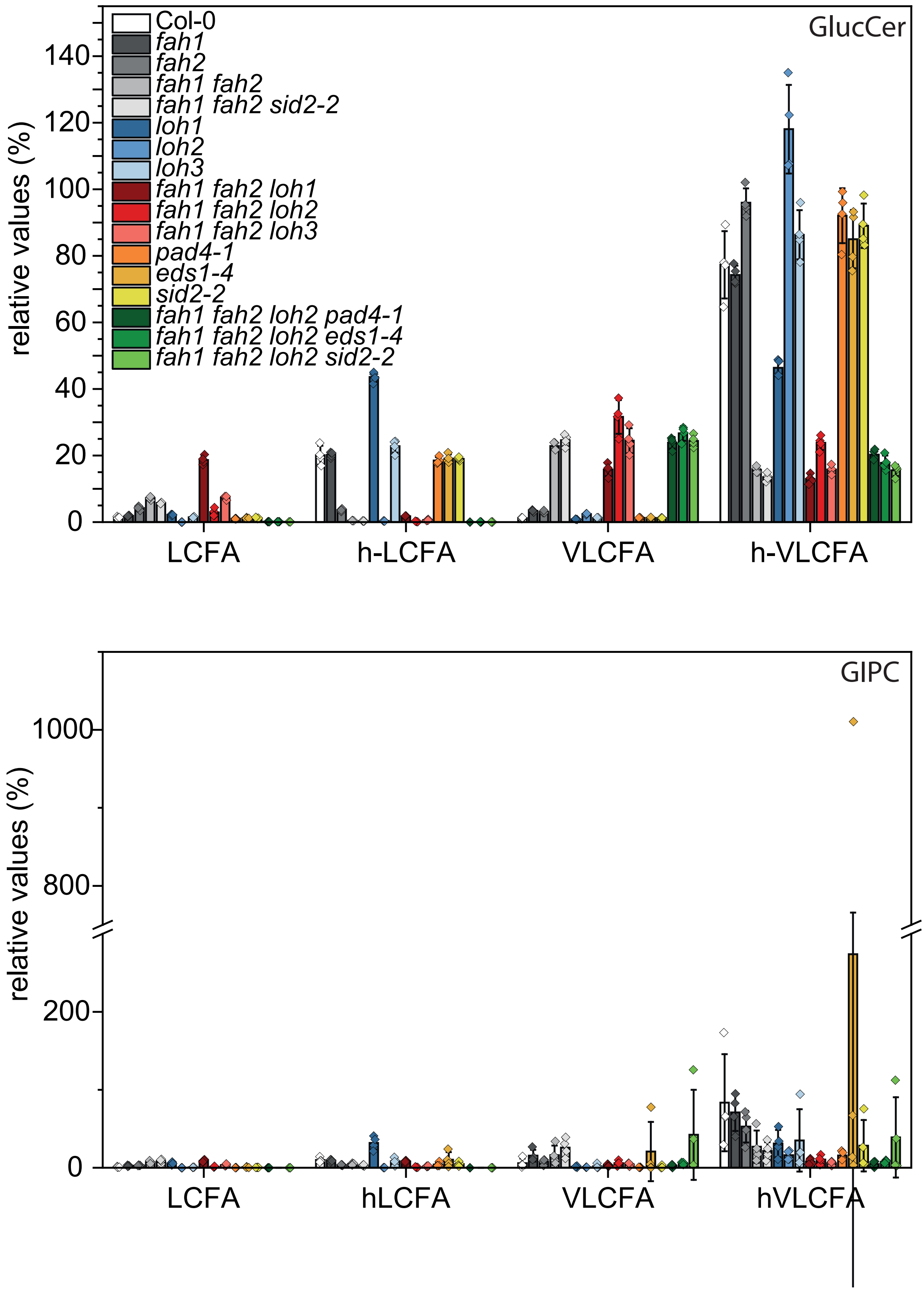

Fig S3 GlucCer (A) and GIPC (B) profiles in crosses of *fah1 fah2* double and *fah1 fah2 loh2* triple mutants with SA synthesis (*sid2-2*) and signaling mutants (*eds1-2, pad4-1*). Rosette leaves of 35-day old plants grown under long day conditions were extracted and analyzed. LCFA, Cers with C16-C18 FA moiety; hLCFA, Cers with C16-C18 α-hydroxylated FA moiety; VLCFA, Cers with C20-C28 FA moiety; hVLCFA, Cers with C20-C28 α hydroxylated FA moiety. Relative values were calculated according to 100 % of wild type peak area of the total GlucCer or GIPC signals. Values represent the mean ±SD of four biological replicates (n=4). The experiment was repeated once with similar tendencies.
