# Supplementary material for: Sphingolipid-Induced Programmed Cell Death is a Salicylic Acid and EDS1-Dependent Phenotype in Arabidopsis Fatty Acid Hydroxylase (Fah1, Fah2) and Ceramide Synthase (Loh2) Triple Mutants
Source: Plant Cell Physiol. 2021 Dec 15;63(3):317–25. doi: 10.1093/pcp/pcab174 (PMC8917834; doi:10.1093/pcp/pcab174)
Supplement: pcab174_Supp [file pcab174_supp.zip › pcp-2021-e-00389-File014.pdf]

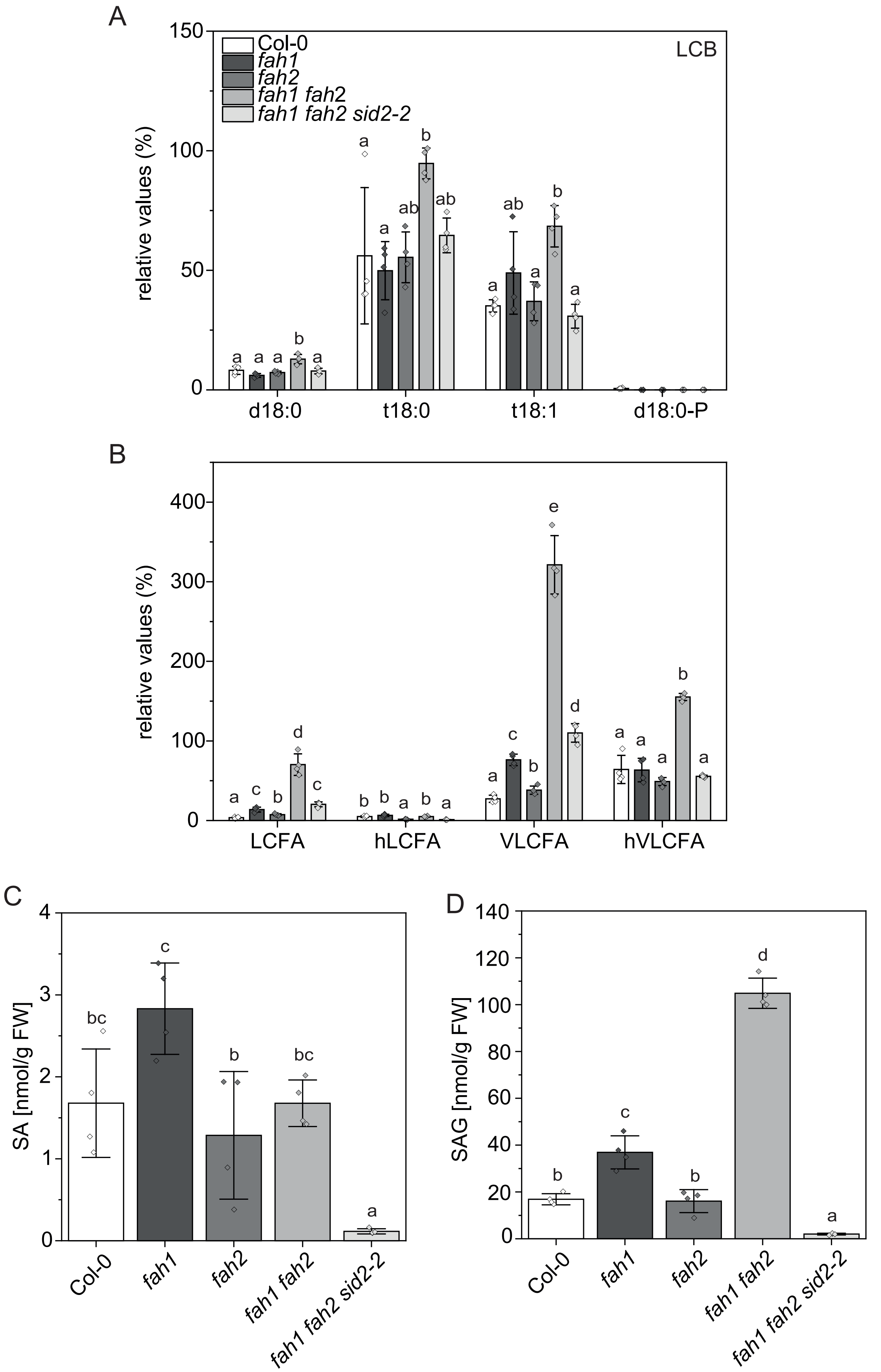

Fig. S4 (A, B) LCB and Cer profiles in crosses of *fah* single and double mutants and the *fah1 fah2 sid2-2* triple mutant. Rosette leaves of 35-day-old plants grown under long day conditions were extracted and analyzed. LCFA, Cers with C16-C18 FA moiety; hLCFA, Cers with C16-C18 α-hydroxylated FA moiety; VLCFA, Cers with C20-C28 FA moiety; hVLCFA, Cers with C20-C28 α hydroxylated FA moiety. Relative values were calculated according to 100 % of wild type peak area of the total LCB or Cer signals. (C, D) SA and SAG contents in 35 day-old rosette leaves of *fah* single and double mutants and the *fah1 fah2 sid2-2* triple mutant. Values represent the mean ±SD of four biological replicates (n=4). Statistical analysis was performed with log-transformed data by one-way analysis of variance (ANOVA) with Tukey's *post hoc* test (P<0.05). The experiment was repeated once with similar tendencies.
