# Supplementary material for: Sphingolipid-Induced Programmed Cell Death is a Salicylic Acid and EDS1-Dependent Phenotype in Arabidopsis Fatty Acid Hydroxylase (Fah1, Fah2) and Ceramide Synthase (Loh2) Triple Mutants
Source: Plant Cell Physiol. 2021 Dec 15;63(3):317–25. doi: 10.1093/pcp/pcab174 (PMC8917834; doi:10.1093/pcp/pcab174)
Supplement: pcab174_Supp [file pcab174_supp.zip › pcp-2021-e-00389-File015.pdf]

Figure S5

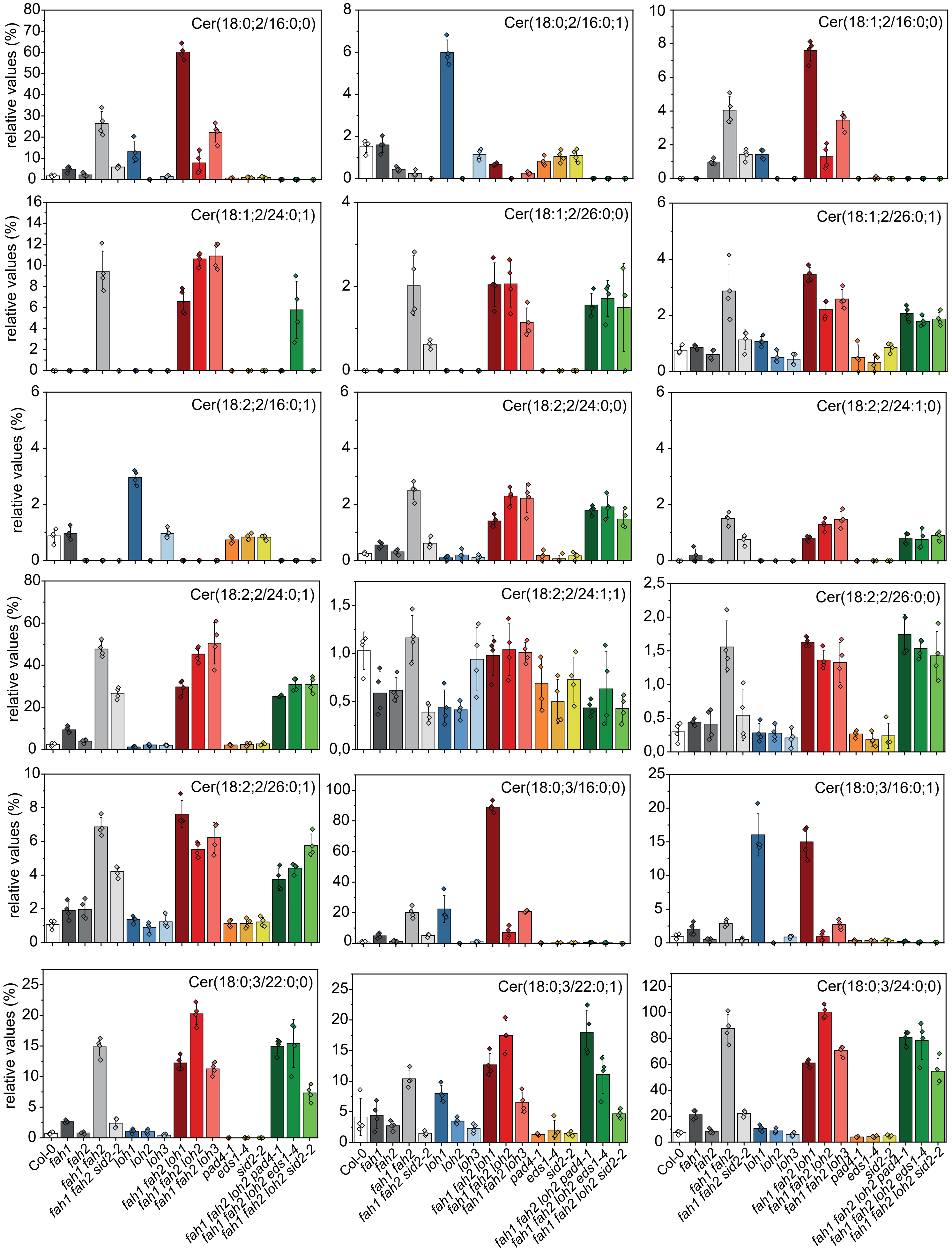
relative values (%)
Cer(18:0;2/16:0;0)
Cer(18:0;2/16:0;1)
Cer(18:1;2/16:0;0)
Cer(18:1;2/24:0;1)
Cer(18:1;2/26:0;0)
Cer(18:1;2/26:0;1)
Cer(18:2;2/16:0;1)
Cer(18:2;2/24:0;0)
Cer(18:2;2/24:1;0)
Cer(18:2;2/24:0;1)
Cer(18:2;2/24:1;1)
Cer(18:2;2/26:0;0)
Cer(18:2;2/26:0;1)
Cer(18:0;3/16:0;0)
Cer(18:0;3/16:0;1)
Cer(18:0;3/22:0;0)
Cer(18:0;3/22:0;1)
Cer(18:0;3/24:0;0)
Col-0
fah1
fah2
fah1 fah2
fah1 fah2 sid2-2
loh1
loh2
loh3
fah1 fah2 loh1
fah1 fah2 loh2
fah1 fah2 loh3
pad4-1
eds1-4
sid2-2
fah1 fah2 loh2 pad4-1
fah1 fah2 loh2 eds1-4
fah1 fah2 loh2 sid2-2

Figure S5

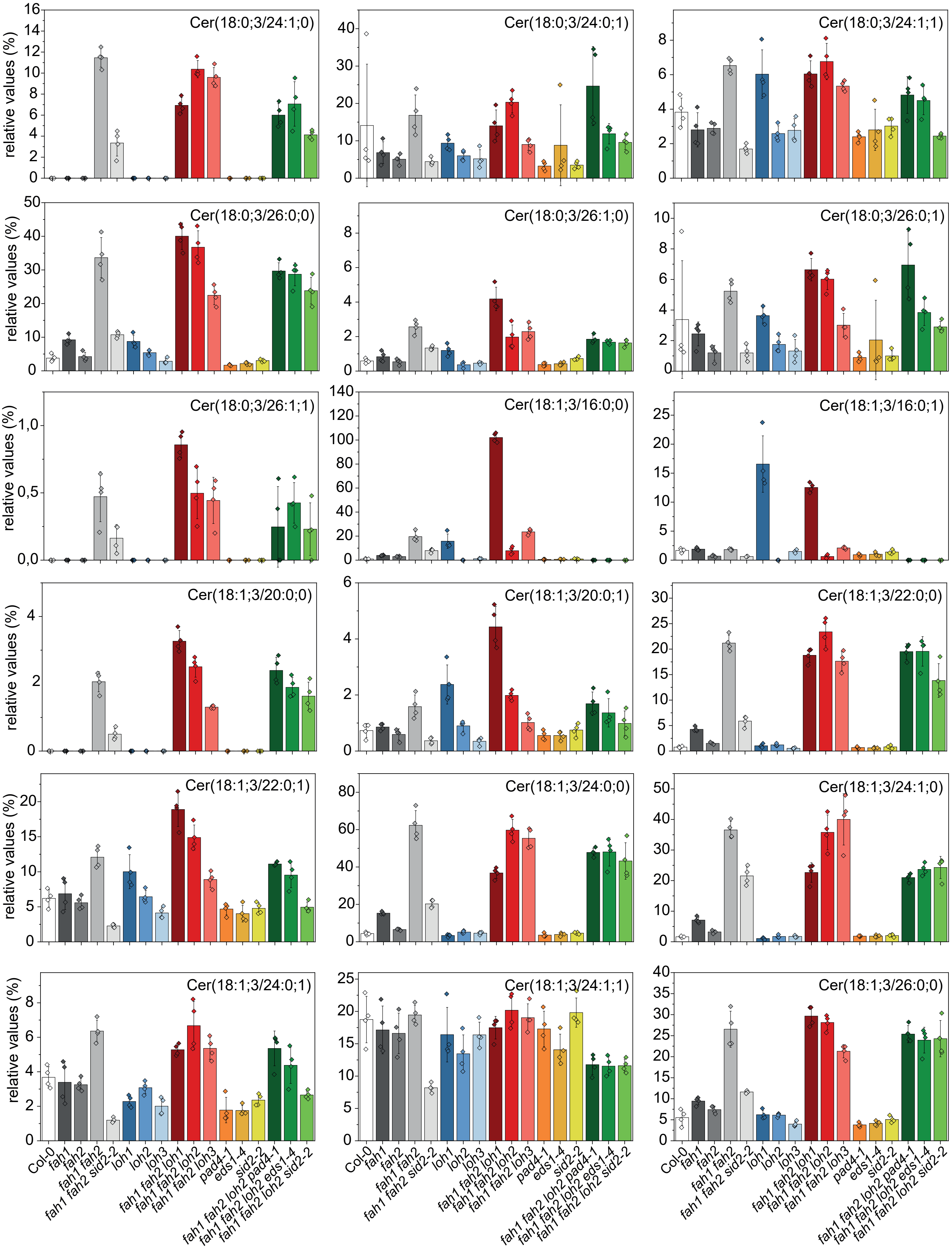
Cer(18:0;3/24:1;0)
Cer(18:0;3/24:0;1)
Cer(18:0;3/24:1;1)
Cer(18:0;3/26:0;0)
Cer(18:0;3/26:1;0)
Cer(18:0;3/26:0;1)
Cer(18:0;3/26:1;1)
Cer(18:1;3/16:0;0)
Cer(18:1;3/16:0;1)
Cer(18:1;3/20:0;0)
Cer(18:1;3/20:0;1)
Cer(18:1;3/22:0;0)
Cer(18:1;3/22:0;1)
Cer(18:1;3/24:0;0)
Cer(18:1;3/24:1;0)
Cer(18:1;3/24:0;1)
Cer(18:1;3/24:1;1)
Cer(18:1;3/26:0;0)
relative values (%)
Col-0
fah1
fah2
fah1 fah2
fah1 fah2 sid2-2
loh1
loh2
loh3
fah1 fah2 loh1
fah1 fah2 loh2
fah1 fah2 loh3
pad4-1
eds1-4
sid2-2
fah1 fah2 loh2 pad4-1
fah1 fah2 loh2 eds1-4
fah1 fah2 loh2 sid2-2

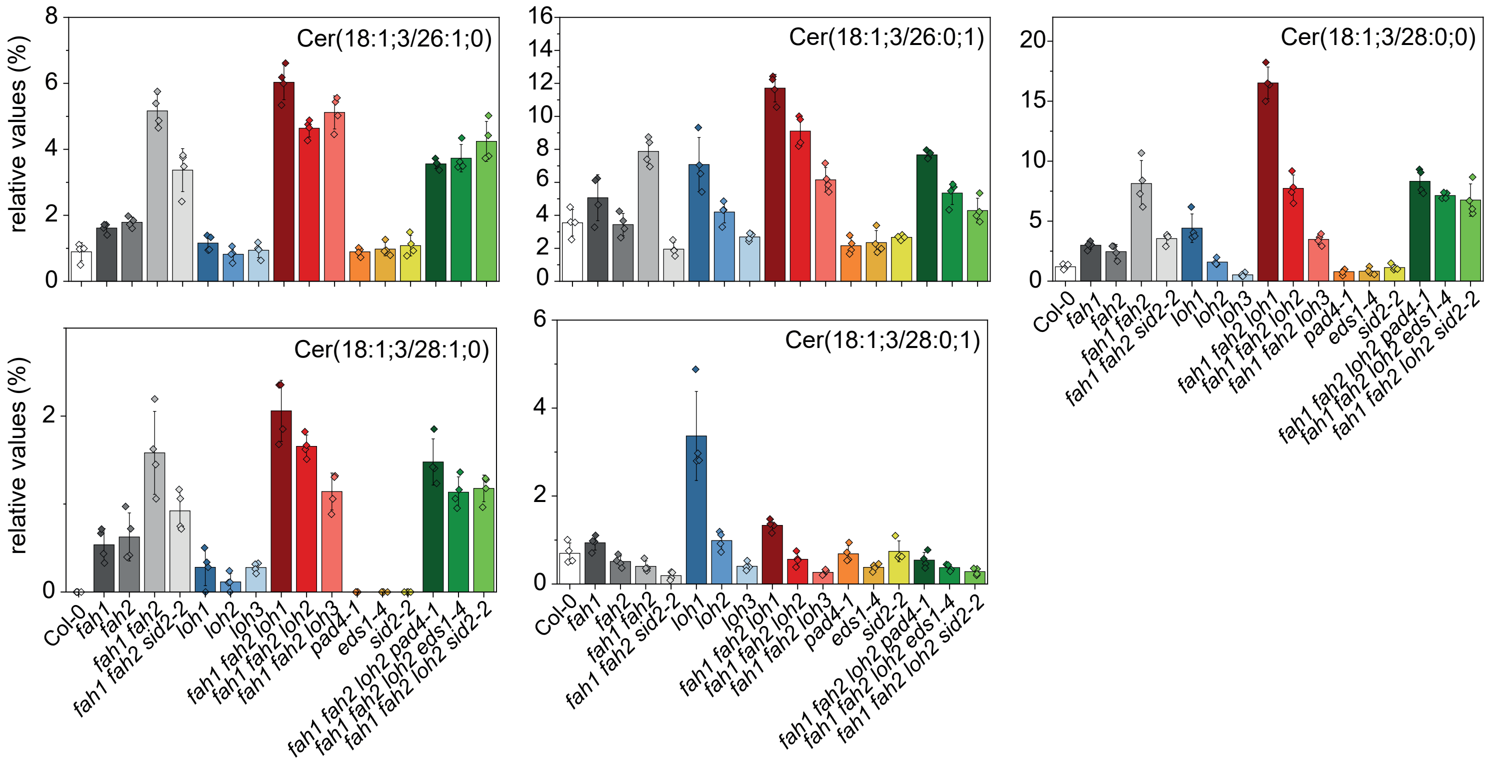


**Fig. S5** Single Cer species in crosses of *fah1 fah2* double and *fah1 fah2 loh2* triple mutants with SA synthesis (*sid2-2*) and signaling mutants (*eds1-2, pad4-1*). Rosette leaves of 35-day-old plants grown under long day conditions were extracted and analysed. Values represent the mean ±SD of four biological replicates (n=4).
